# Vanishing fingers: systemic sclerosis-associated acro-osteolysis

**DOI:** 10.1093/qjmed/hcae156

**Published:** 2024-08-09

**Authors:** H Yamamoto, Y Taniguchi

**Affiliations:** Department of Endocrinology, Metabolism, Nephrology and Rheumatology, Kochi Medical School Hospital, Kochi University, Nankoku, Kochi, Japan; Department of Endocrinology, Metabolism, Nephrology and Rheumatology, Kochi Medical School Hospital, Kochi University, Nankoku, Kochi, Japan

**Figure 1.**
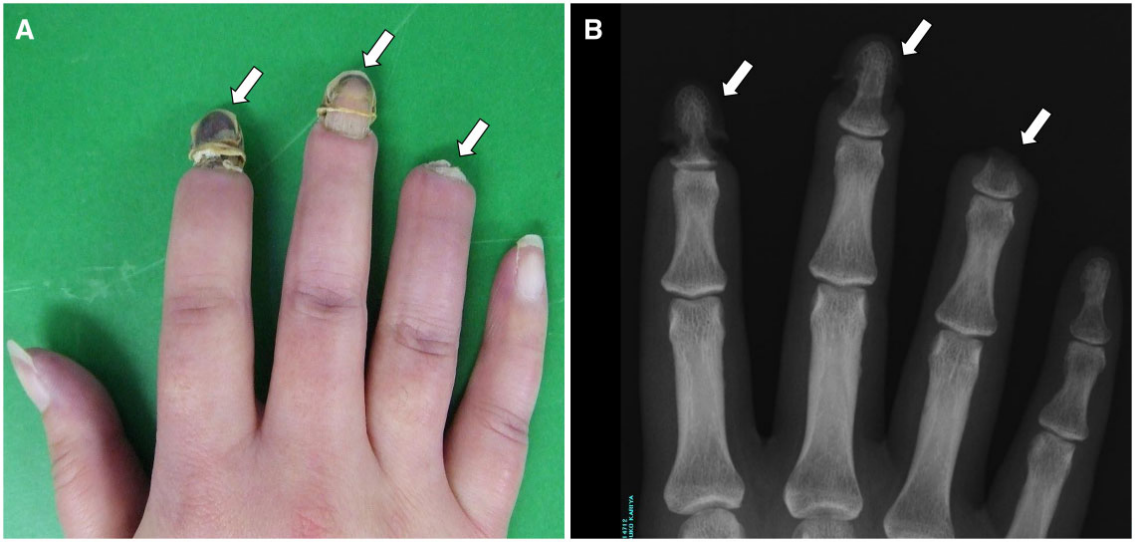
Physical and X-ray findings of systemic sclerosis associated acro-osteolysis.

A 37-year-old woman presented with digital pain and discoloration of 2 years duration. Physical examination revealed Raynaud’s phenomenon, sclerodactyly and digital gangrene (Figure 1A, arrows). The test results for antinuclear antibody and anti-ribonucleoprotein antibody were positive. But, antineutrophil cytoplasmic antibodies, anti-double-stranded DNA, anti-Topo-I, anti-centromere and antiphospholipid antibodies were all negative. Monoclonal protein and cryoglobulin were not detected by immunoelectrophoresis. Notably, a hand X-ray demonstrated osteolysis of the distal phalanx (Figure 1B, arrows). Thus, she was diagnosed with acro-osteolysis due to systemic sclerosis (SSc). Vasodilators, antiplatelet drug and immunosuppressant were initiated, but her clinical condition did not recover. Then, digital necrosis was surgically resected.

Progressive acro-osteolysis is strongly associated with severe digital ischemia and the incidence was reported to be about 9.8 per 100-person-years in SSc patients. On the other hand, after 3 years of follow-up, the rate of progression increased remarkably, resulting in half of the patients developing severe osteolysis [1, 2]. This case reminds readers to consider SSc as a cause of acro-osteolysis.
